# The Demoralising Tendency of Reformatory and Industrial Schools

**Published:** 1896-02-01

**Authors:** 


### Modern Sociology.

### THE DEMORALIZING TENDENCIES OF REFORMATORY AND INDUSTRIAL SCHOOLS.

One of the first fruits of the great wave of revulsion against the harsh and inhuman methods of punishment for petty offences which swept across the land in the earlier years of the present century was the recognition of the advantages of preventive over punitive measures, especially as regarded juvenile offenders. The herding together in prison of hardened and incorrigible criminals of mature years with children who, by reason of their youth and inexperience, could not possibly be deemed to be utterly depraved and hopeless, or who had only been led by chance circumstances into some isolated or trivial act of transgression, was so obviously illogical and unjust that the need of some special and more merciful treatment of them asserted itself beyond all question. Hence the establishment in 1838, by special enactment, of the Parkhurst Reformatory School, and the subsequent extension of the principle by the general Acts of 1854 and 1857, under which most of the existing reformatory and industrial schools were established. These now number 50 of the former and 141 of the latter class, and may fairly claim to take rank as firmly-established national institutions. Since 1854 48,000 children have passed through the reformatory schools, and since 1861 76,000 have been dealt with in the industrial schools. The numbers under detention on December 31st, 1894, were respectively 4,662 and 14,072. With figures like these before us the importance of any question of imperfection of management or administration is obvious, and demands serious attention.

Having in view the immediate object for which the schools were established—the prevention of crime—it must be admitted that they have justified their existence, and contributed in no small degree to the remarkable and steady decrease in the number of convicted criminals attested by the annual returns of H.M. Commissioners of Prisons. The children have been rescued from evil associations and from their own idle and vicious propensities. They have been taught useful trades, to which they have afterwards applied themselves, or they have been encouraged and assisted to emigrate or enlist in the army or navy; whilst in the case of girls they have been trained for and placed out at domestic service or other suitable employment. Out of a total number of 124,615 dealt with from the first, up to the end of 1894 only 3,030 are returned as discharged incorrigible or as having absconded. Satisfactory as these figures undoubtedly are, there is, however, another view of the matter which appears to demand attention. The association together of large numbers of children, all being more or less of vicious, or at least of dubious, antecedents, gives rise to very serious reflections upon the moral consequences likely to ensue, and to raise the question whether the system of classification adopted, and the precautions against moral contamination are, as they ought to be, the best that can be devised. It is true that the reports of the Government Inspector disclose very few instances of libidinous misconduct, or of any serious breaches of the mora law. Most of the offences for which it has been found necessary to inflict punishment appear to be of a mischievous nature. But it must not be inferred on this account that the suggested evil is non-existent; for it is impossible to conceive that the best and most innocent of the inmates of the schools can remain long in daily contact with those of lower moral tone, and with some of the very worst type of character, without detriment. Even the risk of such a thing as this ought to be avoided or minimised by careful and systematic classification; and the absence of any attempt at this appears to be the one great defect of the whole organisation. There is absolutely nothing beyond the statutory distinction between reformatory and industrial schools; the former being intended for the reception of children guilty of felony or other indictable offences, and the latter for those who are charged with minor offences, or who for other reasons come within the scope of the Act. There can be no question that many of the inmates of industrial schools are quite as vicious and depraved as many who have found their way into the reformatories; whilst there is good reason to believe that many of the latter might with propriety be detained in industrial schools. The arbitrary distinction should be modified, if not abolished, and a more rational system adopted. Until two years ago it was a condition precedent to admission to a reformatory school that at least ten days' imprisonment should first be endured, and many a magistrate in passing this hard sentence has lamented the obligation he could not avoid of consigning a child to prison. This remnant of the vindictive spirit of the past was swept away in 1893; and it remains for us to insist upon the changes in the law which may be necessary to avoid or reduce the risk of moral corruption by evil associations. The schools should, themselves, be classified in grades for the reception of inmates of corresponding degrees of depravity or comparative innocence; these characteristics being determined by careful preliminary investigation into the antecedents and the dispositions of the subjects, and not, as at present, entirely by the nature of the offence or the circumstance which happens to form the groundwork of the proceedings against them. The hard and fast principles of bygone days are ill-adapted in most things to the needs of the present, and in none more so than in regard to the subject under consideration.

